# Doloplus-2, a valid tool for behavioural pain assessment?

**DOI:** 10.1186/1471-2318-7-29

**Published:** 2007-12-19

**Authors:** Jacob C Hølen, Ingvild Saltvedt, Peter M Fayers, Marianne J Hjermstad, Jon H Loge, Stein Kaasa

**Affiliations:** 1 Pain and Palliation Research Group, Department of Cancer Research and Molecular Medicine, Faculty of Medicine, Norwegian University of Science and Technology, Trondheim, Norway; 2 Geriatric Section, Medical Department, St. Olav's Hospital, Trondheim, Norway; 3 Department of Public Health, University of Aberdeen Medical School, Aberdeen, UK; 4 Department of Oncology, Ullevaal University Hospital HF Oslo, Norway; 5 Palliative Medicine Unit, Ullevaal University Hospital HF Oslo, Norway; 6 Palliative Medicine Unit, Department of Oncology and Radiotherapy, St. Olav's Hospital, Trondheim, Norway

## Abstract

**Background:**

The Doloplus-2 is used for behavioural pain assessment in cognitively impaired patients. Little data exists on the psychometric properties of the Doloplus-2. Our objectives were to test the criterion validity and inter-rater reliability of the Doloplus-2, and to explore a design for validations of behavioural pain assessment tools.

**Methods:**

Fifty-one nursing home patients and 22 patients admitted to a geriatric hospital ward were included. All were cognitively impaired and unable to self-report pain. Each patient was examined by an expert in pain evaluation and treatment, who rated the pain on a numerical rating scale. The ratings were based on information from the medical record, reports from nurses and patients (if possible) about pain during the past 24 hours, and a clinical examination. These ratings were used as pain criterion. The Doloplus-2 was administered by the attending nurse. Regression analyses were used to estimate the ability of the Doloplus-2 to explain the expert's ratings. The inter-rater reliability of the Doloplus-2 was evaluated in 16 patients by comparing the ratings of two nurses administrating the Doloplus-2.

**Results:**

There was no association between the Doloplus-2 and the expert's pain ratings (R^2 ^= 0.02). There was an association (R^2 ^= 0.54) between the expert's ratings and the Doloplus-2 scores in a subgroup of 16 patients assessed by a geriatric expert nurse (the most experienced Doloplus-2 administrator). The inter-rater reliability between the Doloplus-2 administrators assessed by the intra-class coefficient was 0.77. The pain expert's ratings were compared with ratings of two independent geriatricians in a sub sample of 15, and were found satisfactory (intra-class correlation 0.74).

**Conclusion:**

It was challenging to conduct such a study in patients with cognitive impairment and the study has several limitations. The results do not support the validity of the Doloplus-2 in its present version and they indicate that it demands specific administration skills.

## Background

Pain is common in elderly institutionalized patients, and prevalence rates ranging from 45% to 84% have been reported [1,2]. Cognitive impairment is also common in the same group, and more than 50% of nursing home residents have been found to be cognitively impaired [3,4]. A recent review reported prevalence rates in palliative care patients ranging from 14% to 44%, rising to 90% prior to death [5].

Proper pain assessment is a prerequisite for optimal pain treatment [6], but pain assessment is challenging in cognitively impaired patients. Pain is therefore often overlooked in these patients [3,7-10], leaving them at risk for sub-optimal pain treatment [7,11,12]. When feasible, self-report assessment of pain is regarded as the standard method [9,13]. In patients with mild to moderate cognitive impairment, studies have reported completion rates ranging from 47% to 100% for simple self-report tools such as numerical rating scales and verbal rating scales [3,10]. Ratings of present pain intensity have the highest completion rates, while self-report of other pain dimensions, like location, interference and temporal patterns, is more challenging [10].

Cognitive impairment can make self-report tools for pain assessment invalid and consequently limits their usefulness. Observational assessment of behaviour is an alternative. While self-report tools primarily assess communicative pain behaviours that are under the subject's control, observational tools assess behaviours that are more unconscious or automatic [14]. Behavioural assessment tools are therefore appropriate in subjects with impaired higher mental processes. However, thoroughly validated tools for behavioural assessment are scarce [10,15], and several reviewers have noted the lack of validation of the tools for behavioural pain ratings in the cognitively impaired [2,7,10,15-17]. Although data are limited, a recent review rated the psychometric aspects of 12 behavioural assessment tools according to several quality judgement criteria. Five tools (in English versions) received a satisfactory evaluation of validity and reliability [17]: the Abbey Scale [18], the Pain Assessment for the Dementing Elderly (PADE) [19], the Pain Assessment in Advanced Dementia Scale (PAINAD) [20], the Pain Assessment Checklist for Seniors With Limited Ability to Communicate (PACSLAC) [21], and the Doloplus-2 [22]. The review concluded by recommending the PACSLAC and the Doloplus-2, stating that they seem promising but required further testing [17].

The Doloplus, launched by Bernard Wary in 1992/93, was originally a 15-item clinical tool for proxy rating of pain in elderly patients with cognitive failure [22,23]. It was based on a tool for behavioural assessment of pain in children with neoplastic disease (Douleur Enfant Gustave Roussy scale) [22,23]. In 1995, the Doloplus was refined by a French/Swiss network of geriatricians, resulting in the present ten-item version (Doloplus-2 [22]). A Doloplus-2 assessment is performed by a proxy-rater who observes the subject and evaluates the presence of ten pain-related behaviours from 0 to 3 – representing increasing presence of the behaviour [22,24]. These include: verbal complaints, facial expressions, protective body postures, protection of sore areas, disturbed sleep, functional impairment in activities of daily living (washing and dressing, and general mobility), psychosocial reactions such as behavioural problems, and changes in communication or social life. Authors of the Doloplus-2 suggest a cut-off score of 5 out of 30, representing possible pain being present [22,23], but this has not been empirically validated.

Despite a shortage of validation studies published in international journals and despite a call for thorough validation [25], including information on inter-rater reliability [17], the French version of the Doloplus-2 is in widespread clinical use in France and Switzerland [23]. This prompted us to undertake a Norwegian pilot validation study in 2004, in which we evaluated 59 patients who were institutionalized in nursing home units for the demented [24]. While well established protocols are available for the validation of self-report based assessment tools there is no consensus on how to validate tools for observational assessment. The objective of this pilot study was to translate the Doloplus-2 from French into Norwegian, to test the translation, explore the user-experiences, and evaluate the criterion validity of the Doloplus-2. The aim was to test the Doloplus-2 in patients who were unable to self-report and therefore we compared nurses' Doloplus-2 scores to pain scores (pain criterion) given by pain experts who examined these patients (R^2 ^= 62%). The results demonstrated satisfactory criterion validity in some domains. The Doloplus-2 item for *facial expressions *was the most informative, while the item for *social life *contributed least. All the three items forming the psychosocial domain (Communication, Social life, and Behavioural problems) were reported as problematic to conceptualize and contributed marginally to explain the expert pain score [24]. These results were supported by a recent study that evaluated the psychometric properties of the Doloplus-2, and two other tools for behavioural pain assessment, by comparing observer based pain scores from two independent raters [26]. This study found low congruent validity in the Doloplus-2, it questioned the validity of the psychosocial domain, and its clinical usefulness was evaluated as moderate by the participating nurses. The authors acknowledged that the study design was less adequate for exploring the psychometric properties of the Dololplus-2 compared to the other tools and they requested more studies on the validity and intra- and inter-reliability of the Doloplus-2 [26].

Based on the previous results and our experiences with the use of a pain expert as a criterion for pain, a new study was launched in order to further study the psychometric performance of the Doloplus-2. The objectives of the present study were to:

1. Assess the criterion validity of the Doloplus-2 in patients who are unable to self-report pain due to cognitive failure.

2. Test the inter-rater reliability of a pain expert's ratings (used as criterion).

3. Evaluate the inter-rater reliability of the Doloplus-2 by comparing the results from different and independent administrators.

## Methods

### Subjects

The subjects were a convenience sample of 73 consecutively recruited patients from two nursing homes (N = 51) and from the Section of Geriatrics at St. Olav's University Hospital (N = 22) in Trondheim, Norway. As previous publications had demonstrated pain to be prevalent in regular nursing homes and in geriatric hospital units [3,27], these were approached under the assumption that painful somatic conditions would be prevalent. The patients should be unable to self-report pain due to cognitive impairment based upon the nurses' clinical evaluation of the patients. Pain was defined as and limited to somatic pain, i.e. a symptom generally relieved by analgesics, and consequently excluding what the pain expert interpreted as existential pain.

### Baseline characteristics

Cognitive function was assessed by the Mini Mental State Examination (MMSE) [28,29] administered by either a ward nurse or a medical student. The MMSE rates the level of cognitive function on a scale from 0–30. Patients with scores from 30-21 are regarded as normal to mildly cognitively impaired, scores from 20-11 denote moderate cognitive impairment, and patients scoring 10-0 are severely cognitively impaired [30]. The MMSE was performed within the same week as the main data collection. At one nursing home ward the MMSE was performed within a month (N = 10). Due to the patients' stabile conditions this was regarded as appropriate. Ability to perform activities of daily living (ADL) was evaluated by a nurse familiar with the patient, using the Barthel Index [31]. This tool describes the ability to perform ADL on a scale from 0–20. Barthel index scores from 20-15 indicate independence to mildly disabled ADL function, 14-10 indicate moderate disability, while a score of 9-0 indicate that the patient is severely to very severely disabled [32]. The Barthel Index was completed within a week of the Doloplus-2. The MMSE and Barthel Index measures were used to provide a baseline characteristic of the patients' status. Information regarding patients' use of analgesics was not recorded. Because our aim was to test if the Doloplus-2 could assess pain in those who experienced pain, it was not regarded necessary to know if a low level of pain behaviours were caused by adequate treatment or lack of pain.

### Criterion validity of the Doloplus-2

The Doloplus-2 [22,24] is composed of ten items distributed on three domains: somatic, psychomotor and psychosocial. The somatic domain has five items, while the psychomotor and psychosocial domains have two and three items, respectively. Each item has four response alternatives, and is scored 0 for normal behaviour, through to 3 for high levels of pain-related behaviour. Thus the total Doloplus-2 score ranges from 0–30.

The Doloplus-2 was administered by trained enrolled nurses, or registered nurses who were familiar with the patient. The attending daytime nurse completed the Doloplus-2 registration after consulting with the other personnel who had been involved with the patient during the past 24 hour period. Pain in the Doloplus-2 was registered according to the instructions for Doloplus-2 and recorded once for each patient, usually between noon and 3 p.m. [22].

In line with psychiatric methods for observational assessment and diagnoses in cases where an objective measure is inaccessible a clinical expert statement was used as the criterion for pain [33-35]. A pain specialist nurse (pain expert) from the National Centre of Expertise for Pain and Complex Disorders at St. Olav's University Hospital of Trondheim made a single evaluation of each patient's pain level on an eleven point Numerical Rating Scale (NRS-11) from zero (no pain) to ten (worst imaginable pain). Each patient was ascribed two pain intensity scores, one for pain in movement and one for pain at rest. These scores were used as the pain criterion. The pain evaluation was performed the same day as the Doloplus-2 assessment, usually between noon and 4 p.m. The expert's evaluation made use of information from the medical record, reports from nurses and patients (if possible) about pain during the past 24 hours, and a clinical examination. The clinical examination comprised observation of the patient during rest and activity, and examination of common trigger points for pain. Both the expert's pain score and the nurses' Doloplus-2 scores were based upon the same time interval and all assessors had access to information about the patient's medical condition during the past 24 hours. The expert was blinded from the Doloplus-2 administrators' assessment, and vice versa.

As a validation of the evaluations performed by the pain expert, two geriatricians with expertise in pain presentation in demented patients observed the pain expert while he evaluated 15 consecutive patients. Without discussing the patients with the pain expert, the two geriatricians independently rated the patients' pain using NRS-11. Their ratings were later examined for degree of association with the expert's ratings.

### Inter-rater reliability

Inter-rater reliability of the Doloplus-2 was assessed in 16 patients consecutively included at the Section of Geriatrics at St Olav's University Hospital. A geriatric specialist nurse (GN) and an enrolled nurse evaluated each patient at the same day and blinded from each other. The GN assessed all patients, while a team of six different enrolled nurses made the second assessment.

**Figure 1:**
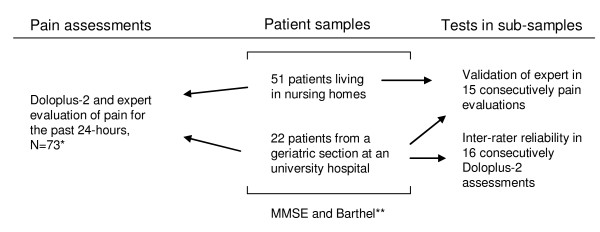
**Study procedure**. *Performed the same day, usually performed between noon – 3 p.m. **The MMSE and Barthel were performed within the same week as the pain assessments except from ten MMSE that was performed within a month.

See Figure 1 for overview of the study procedure.

### Analyses

Univariate regression analyses were performed in order to analyse how well the Doloplus-2 predicted the expert pain score (R-squared), and to analyse the contributions of each of the ten items. Since the Doloplus-2 score maximizes pain by adding the scores of all items, we chose to compare with the highest of the pain expert's scores. The pain-in-movement score was higher than the pain-at-rest score in all patients and consequently used as the pain criterion.

Association between the expert's pain ratings and the two geriatricians' ratings and the inter-rater reliability of the Doloplus-2 were evaluated with intra-class correlation coefficients. All analyses were performed by the SPSS statistical software version 13.0 (SPSS Inc., Chicago, USA).

### Ethics

The Regional Committee for Medical Research Ethics approved the study. As recommended by the committee, written informed consent was not obtained from the patients due to their cognitive impairment. Instead, the patient's nearest relative was informed, both in writing and orally, and asked to give consent. Eligible patients were informed orally and asked if they would participate before the administration of the MMSE and the pain expert evaluation. Patients were not to be included if they or their relative declined participation, but no one did.

The constructors of the Doloplus-2 have approved our use of the tool.

## Results

### Baseline characteristics

Seventy-three patients were approached and all were included. The mean age of the sample was 84 years (Table 1), and 74% were female. The median MMSE score was 10 (Table 1). Two subjects died before the MMSE assessment, and seven were not assessed as they moved to another nursing home before the MMSE assessment. These patients were included in the pain analyses, but excluded from the MMSE calculations. The Barthel Index scores had a median value of 9 (Table 1).

**Table 1:** Distribution frequencies of background variables

**Age **(mean 84)		Numbers (N = 73)
	69–79 years	19 (26%)
	80–90 years	37 (51%)
	> 90 years	17 (23%)

**MMSE-Score **(median 10)*		

Severely cognitively impaired (CI)	0–10	32 (50%)
Moderately CI	11–20	23 (36%)
Mildly CI to normal	21–30	9 (14%)

**Barthel Index-Score **(median 9)		

Very severely to severely disabled	0–9	41 (56%)
Moderately disabled	10–14	18 (25%)
Mildly disabled to independent in ADL	15–20	14 (19%)

*9-missing

### Validity of the Doloplus-2

The expert rated seven patients ≥ 4 for pain-in-movement (moderate-to-severe pain), 40 were rated 1–3, and 26 were rated as without pain. In all patients, the pain-in-movement score was equal to or higher than the pain-at-rest score. The association between the pain expert's ratings and the two geriatricians' ratings (N = 15) was estimated with an intra-class correlation of 0.74 with a 95% confidence interval from 0.5 to 0.89.

The mean Doloplus-2 score was 7.47 (SD = 5.08) with a range from 0–22. Five patients received a Doloplus-2 score of zero. Among these, three were also rated with no pain by the expert, while the other two received a score of zero at rest and two in movement.

**Figure 2:**
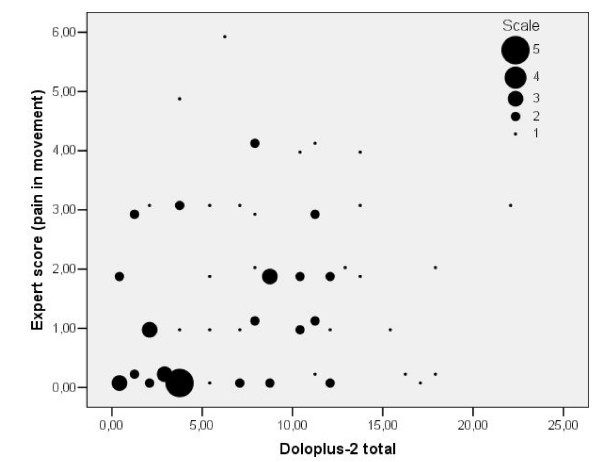
**The relationship between expert ratings and Doloplus-2**. The scatter plot demonstrates the relationship between the expert's pain score (NRS-11) and the Doloplus-2 score (0–30) in all 73 patients.

The regression analysis of the Doloplus-2 scores against the expert scores produced an R^2 ^of 0.023, implying poor criterion validity of the Doloplus-2 in this data set (Figure 2).

To explore the data more closely we analysed each study site separately. No significant results were obtained while looking at the complete data from the three sites; however, association was found between the pain expert and the geriatric expert nurse (GN) who administered the Doloplus-2 in 16 patients in the Section of Geriatrics, with an R^2 ^of 0.54.

Univariate regression analyses of the different Doloplus-2 items (full sample) showed small but significant relationships between the Doloplus-2 item for *protective body postures at rest *and the expert's pain-in-movement score (R^2 ^= 0.12, p = 0.003) and for the Doloplus-2 item *pain complaints *and the expert's pain-at-rest score (R^2 ^= 0.13, p = 0.002).

### Inter-rater reliability

The intra-class correlation for inter-rater reliability of the Doloplus-2 administrators was 0.77, with a 95% confidence interval of 0.47 – 0.92.

## Discussion

Herr *et al*. (2004) called for extensive testing of the Doloplus-2 to provide sufficient details on which to base sound judgment of the tool, and a recent review questioned both the specificity of the Doloplus-2 and the nurses' competence for scoring and interpreting the results [17]. The present study failed to confirm a valid relationship between the expert's ratings of pain and the Doloplus-2 scores in a sample of 73 cognitively impaired patients, even though the inter-rater reliability of the Doloplus-2 seemed to be satisfactory. These results differ from those of our previous pilot validation study [24], in which acceptable criterion validity was demonstrated when comparing Doloplus-2 against expert ratings.

We acknowledge several limitations in the present study. The samples sizes were small as indicated by the confidence intervals for the inter-rater analyses and the majority of the subjects were female (74%). Use of analgesics was not recorded. Analgesic efficacy might fluctuate throughout the day and information on the use of analgesics could have provided valuable baseline information. However, since all patients were evaluated on the basis of the full 24-hour period any potential bias from analgesics should be equivalent in both the expert and in the Doloplus-2 assessments.

The use of a pain expert's rating as a pain criterion is disputable as it may be questioned whether this represents a valid criterion. In line with psychiatric methodology for cases where no obvious gold standard exists, we used an expert-evaluation of the patients as the pain criterion. We tested the expert's performance in a sub-sample (N = 15) and found satisfactory inter-rater reliability between the expert and the two geriatricians. The low end of the confidence interval for the inter-rater reliability indicates that despite the small sample size of 15 evaluations the agreement is satisfactory.

It is probably an advantage for an expert-rater to know the patients. In the pilot study, the physicians responsible for the patients' treatment acted as the expert [24]. It is possible that the lack of association between Doloplus-2 and the expert's rating in the present study may partly be due to the use of an external pain expert who was unfamiliar with the patients instead of one who was familiar with the patients and the staff. The expert evaluated the presence of pain at rest and in movement. The Doloplus-2 does not distinguish between rest and movement, but adds all scores together. We decided to use the higher obtained of the two expert scores. This was without exception the pain-in-movement score. We do not suggest that the Doloplus-2 is designed to measure only pain in movement, but we believe that the expert's pain-in-movement score is the best indicator of pain in these patients.

Five patients were rated with a Doloplus-2 score of zero, as opposed to 26 in the expert's ratings. In order to discuss the discrepancy between Doloplus-2 and expert score, the pain expert, the GN and the Doloplus-2 administrators at one nursing home were consulted. It was impossible to know whether the pain expert identified false negative pain cases or whether the Doloplus-2 identified false positives. A general conclusion was that Doloplus-2 assessment in many of these patients was perceived as difficult. In patients who had high Doloplus-2 scores and low expert scores, clinical examinations revealed that the patients' discomfort seemed frequently related to grief, depression, anxiety and/or agitation rather than to somatic pain. This might suggest that the Doloplus-2 identifies patients with pain who may not have somatic pain (i.e. false positives), on the other side we can not out rule that this is a result from the pain expert underrating pain. The Doloplus-2 administrators were instructed to give positive scores on items only if changes in behaviour were suspected to be pain-related. In practice however, they were not able to evaluate this. As a result, they may have given positive scores on behavioural changes probably related to other causes than pain. This illustrates some well-known difficulties in pain assessment among patients with Behavioural and Psychological Symptoms of Dementia (BPSD) who are not able to describe their problems thoroughly and who may also have atypical symptom presentation [17,36]. The expert performed a comprehensive evaluation of the patient, but his pain ratings focused on what he judged as somatic pain intensity. The Doloplus-2 approaches pain multidimensional and this difference can partly explain some of the disagreement between expert and Doloplus-2. A concern for the validity of the expert judgments could be the use of patient charts information to inform their judgments. Presumably, this would allow access to information about physical pathology. There is only a marginal correlation between physical pathology and self-report of pain in people able to self-report. Inferring pain from this information can be questionable. However, the pain expert has several years experience from work at the National Centre of Expertise for Pain and Complex Disorders at St. Olav's University Hospital of Trondheim. A majority of the patients coming to this clinic has pain that does not have an identifiable basis in physical pathology. In the study design we wanted to give the expert access to all available information to minimize the chances of underestimating pain. He evaluated the different sources of information towards each other and we are confident in that he did not underrate pain due to lack of information on physical pathology in the charts. Instead, it may strengthen the expert evaluation that the expert was informed about the patients' diagnoses of possible painful chronic conditions.

Analyses indicated that competence in geriatrics improved the validity of the Doloplus-2 assessment. The Doloplus-2 scores had higher correlations with the expert's pain ratings in a small sub-group in which the Doloplus-2 was administered by a specialist GN. This finding was in concordance with the pilot study, where the Doloplus-2 administrators had higher skills than the administrators in the present study, as all assessments were made jointly by an enrolled nurse/registered nurse in cooperation with a fully trained final-year medical student [24]. Thus, it may be hypothesized that valid Doloplus-2 administration and interpretation demand training in geriatrics and knowledge of pain presentation in cognitively impaired patients. Analyses of the sub-group with the GN resulted in a similar pattern of items contributing in explaining the expert's pain score to that found in the pilot study [24]. The items for complaints, disturbed sleep, functionality during washing/dressing, and facial expressions explained most of the expert score, while the three psychosocial items explained close to nothing. The Doloplus-2 was originally developed for pain assessment in children and the inclusion of the psychosocial items may come from this origin. Based on results from Zwakhalen et al. (2007) and our two studies we suggest that the psychosocial domain could be removed from the Doloplus-2.

Some of the patients could provide limited information about pain at the moment and this was demonstrated during the expert's clinical examination and during the morning sessions, while the nurses wash and dress the patients, which caused some patients to express pain complaints, which then again lead to positive expert pain score and positive score on the Doloplus-2 item about pain complaints. The subjects' self-report was consequently taken into account when it was available. Future studies could try a simple verbal rating scale for self-report of pain intensity in some patients and use this in a combination with other criterions.

Reports have shown that pain is frequent, under-recognised and under-treated in nursing homes. Therefore we approached patients at regular nursing home units and at a geriatric department in order to include patients with higher levels of pain than in the pilot study [24]. As expected the MMSE scores and the Barthel Index demonstrated that the study population was cognitively impaired and dependent on care. However, unexpectedly it turned out that the sample had lower average levels of pain, as rated by the experts than, the sample in the pilot study. Low levels of pain have surprised other researchers in the field [36]. Thus, the present study also failed to provide data about the performance of Doloplus-2 in patients with severe pain.

To validate a tool is a long process and solid conclusions regarding the validity of the Doloplus-2 cannot be reached on the basis of our two studies. Through our studies we have established some experience in the design of studies for such validations. Future studies should include some patients with known painful diagnoses like patients with post operative hip-fractures. It will also be most valuable to have more than one pain criterion to test for agreement, in those where self-report is invalid. Pain experts could be used to establish a criterion. The use of more than one expert, blinded or unblinded, in each patient will strengthen the study. Test treatment with analgesics in patients with suspected pain and use of other behavioural tools and a verbal rating scale for present pain intensity may be valuable amendments.

The lack of agreement between expert and Doloplus-2 might reflect a common challenge for pain measurement in cognitively impaired by the use of behavioural assessment tools. Other tools recommended for use in these patients Abbey Scale [18], PADE [19], PAINAD [20], and PACSLAC [21] have obvious similarities to the Doloplus-2. All tools cover facial expressions, abnormalities in body postures/movements like guarding sore areas, impaired movement and verbal expressions. These tools are constructed for administration by health care providers, but to our knowledge none of them claim any criteria with regard to the administrators' competence. All tools include domains that are not only affected by pain. The inclusion of BPSD increases the pain sensitivity in these tools, but the specificity decreases. The consequence may be that comprehensive training of administrators and high administration skills is needed. The brief Checklist of Nonverbal Pain Indicators (CNPI) [37] is another interesting behavioural pain assessment tool, as it covers those parts of the Doloplus-2 that performed most successfully in our studies, but it needs further validation [17]. The CNPI may be an alternative that should be thoroughly tested before finally judged.

## Conclusion

Based on the results from our two studies combined, we recommend the use of more than one pain criterion. Pain experts can be used as one of these, especially in patients that have no or limited ability to self-report. A combination of pain experts, other behavioral pain assessment tools, a verbal rating scale for self-report of present pain intensity and test-treatment with analgesics could constitute a promising pain criterion in future studies. The present study does not support the criterion validity of the Doloplus-2 as a clinical pain assessment tool in its present version. The results indicate that there seems to be a need for systematic training of the administrators before the instrument can be of clinical use.

## Competing interests

The author(s) declare that they have no competing interests.

## Authors' contributions

JCH, IS, PMF and SK contributed to the design of this study. JCH and IS organized and performed the data-collection. JCH, PMF and JHL performed the statistical analysis. All authors participated in interpretation of the data, drafting the manuscript and all read and approved the final manuscript.

## Pre-publication history

The pre-publication history for this paper can be accessed here:


